# The associations between personality traits and quality of life, satisfaction with life, and well-being over time in people with dementia and their caregivers: findings from the IDEAL programme

**DOI:** 10.1186/s12877-023-04075-x

**Published:** 2023-06-06

**Authors:** Anna Hunt, Anthony Martyr, Laura D. Gamble, Robin G. Morris, Jeanette M. Thom, Claire Pentecost, Linda Clare

**Affiliations:** 1 Centre for Research in Ageing and Cognitive Health, University of Exeter Medical School, Exeter, UK; 2 Population Health Sciences Institute, Newcastle University, Newcastle, UK; 3 Institute of Psychiatry, Psychology and Neuroscience, King’s College London, London, UK; 4 School of Health Sciences, The University of Sydney, Sydney, Australia; 5 NIHR Applied Research Collaboration South-West Peninsula, Exeter, UK

**Keywords:** Longitudinal, Alzheimer’s, Carer, Personality trait, Quality of life

## Abstract

**Background:**

Cross-sectional evidence indicates that certain personality traits may influence how well people with dementia and their caregivers are able to live alongside the condition. However, no studies to date have explored these associations longitudinally. The present study aimed to explore whether each of the Five-Factor personality traits were associated with change over two years in perceptions of ‘living well’ for people with dementia and their caregivers. ‘Living well’ was conceptualized as a composite of quality of life, satisfaction with life, and subjective well-being.

**Methods:**

Data were analyzed from 1487 people with dementia and 1234 caregivers who took part in the IDEAL cohort. Participants were categorized into low, medium, and high groups for each trait using stanine scores. Latent growth curve models investigated associations between these groups and ‘living well’ scores for each trait at baseline and at 12 and 24 months. Covariates included cognition in people with dementia and stress in caregivers. A Reliable Change Index was calculated against which to evaluate changes in ‘living well’ scores over time.

**Results:**

At baseline, neuroticism was negatively associated with ‘living well’ scores for people with dementia, while conscientiousness, extraversion, openness, and agreeableness were positively associated. For caregivers, neuroticism was negatively associated with ‘living well’ scores at baseline while conscientiousness and extraversion were positively associated. ‘Living well’ scores were mostly stable over time with no influence of personality traits on observed changes.

**Conclusions:**

Findings suggest that personality traits, particularly neuroticism, have a meaningful impact on how people with dementia and caregivers rate their capability to ‘live well’ at baseline. Over time ‘living well’ scores for each personality trait group were largely stable. Studies utilizing longer follow-up periods and more appropriate measures of personality are needed to corroborate and extend the findings of the present study.

**Supplementary Information:**

The online version contains supplementary material available at 10.1186/s12877-023-04075-x.

## Background

Dementia is a heterogeneous condition associated with progressive decline in cognitive, social, and functional abilities. Over 50 million people live with dementia globally [1], with many residing in their own homes and relying on family or other unpaid caregivers for most of their daily care [2]. This figure is set to rise as population aging continues to accelerate and, while some medications ameliorate dementia symptoms, there is currently no cure or treatment to alter the course of dementia. In this context, it is unsurprising that particular attention has been paid to finding ways of supporting people with dementia and their caregivers to ‘live well’ with the condition.

Theoretical frameworks indicate the potential importance of intrinsic resources in influencing the ability of people with dementia and their caregivers to ‘live well’. For example, Kitwood conceptualized the experience of dementia as an interplay between a person’s neurological impairment, personal psychology, health, and social context [3]. Furthermore, the stress process model of caregiving outcomes [4] highlights the role of both internal (e.g., coping repertoires, belief systems, mastery, and self-esteem) and external (e.g., social support and integration) factors in buffering the negative impact of stress, thus explaining why caregivers in seemingly similar situations can experience caregiving differently. However, these frameworks do not explicitly emphasize how outcomes for either people with dementia or their caregivers may be influenced by personality, which can be viewed as the relatively stable patterns of thoughts, feelings, and behaviors which characterize an individual [5]. According to the widely adopted Five-Factor Model [6, 7], personality comprises five traits: neuroticism, extraversion, conscientiousness, openness, and agreeableness. In combination these five traits represent personality at the broadest level and as such they are not strictly orthogonal, with small intercorrelations being reported [8]. Importantly, each trait is also likely to contribute to the presentation and interaction of the other traits. Furthermore, a wealth of research has established that personality traits change, albeit modestly and in relatively systematic ways, across the life course. While there is some individual variation, the most pronounced changes seem to occur in young adulthood; commonly reported changes in this age group include increases in mean levels of conscientiousness and agreeableness and decreases in mean levels of neuroticism [9, 10]. There is generally greater stability in midlife, with small declines reported in mean levels of most traits after age 65 [9–12]. If there is an association with ‘living well’ then personality, or individual personality traits, could help identify people with dementia and caregivers potentially more vulnerable to adverse outcomes and inform personalized care planning and interventions. Indeed, a recent meta-analysis suggested that personality traits may be modifiable through intervention, with the traits of neuroticism and extraversion showing the most change [13].

‘Living well’ is a multifaceted and highly individual concept which can be indexed through evaluations of quality of life (QoL), satisfaction with life, and subjective well-being [14, 15]. In the general population, there is evidence for a strong relationship between personality traits and indicators of ‘living well’ [16, 17]. However, equivalent research in people with dementia and their caregivers is lacking. The majority of personality research related to dementia explores the association between personality traits and dementia risk. A recent meta-analysis reported robust associations between both high neuroticism and low conscientiousness and increased dementia risk [18]. A small number of studies have also explored personality changes following dementia diagnosis using current (post-diagnosis) and retrospective (pre-diagnosis) informant personality ratings. In these studies, increases in neuroticism and decreases in conscientiousness and extraversion are commonly reported by caregivers of people with dementia, with conscientiousness showing the largest change [19, 20]. Furthermore, one prospective study that collected informant ratings of extraversion in people with dementia annually for up to four years found that extraversion declined as dementia severity increased [21], suggesting that personality may change throughout the course of dementia.

In dementia caregiving research, predominantly cross-sectional studies have explored the relationship between personality traits and potential indicators of ‘living well’. For example, neuroticism has been associated with higher perceived stress [22, 23] and burden [24], increased self-reported symptoms of anxiety [25], poorer perceived health-related QoL in the mental health domain [5], increased self-reported depressive symptoms both cross-sectionally [24] and longitudinally [26], poorer perceived physical health [27], and lower perceptions of positive social support [28]. In contrast, caregivers who score high in conscientiousness, extraversion, agreeableness, and openness report lower perceived stress, burden, and depressive symptoms [23, 24]. There is, however, a need to supplement the overwhelming focus in caregiver personality research on specific outcomes, such as stress or burden, with a broader emphasis on ‘living well’. This is because, using stress as an example, caregivers may respond or adapt to stress by mobilizing social support, utilizing relaxation techniques, and/or seeking professional help. Caregivers may therefore still feel they are ‘living well’ despite the stresses of the caregiving role. Using broader measures of ‘living well’ marks a shift away from a focus on symptoms and difficulties towards a more empowering approach that captures the multi-faceted nature of living with dementia [15].

Only a few cross-sectional studies have explored the relationship between personality and broader ‘living well’ measures in people with dementia and their caregivers. In people with dementia, one study found that higher conscientiousness was associated with better QoL when statistically considered individually but not when considered alongside other factors [29]. However, the impact of the other four traits was not explored. Additionally, studies using cross-sectional data from the Improving the experience of Dementia and Enhancing Active Life (IDEAL) cohort found that neuroticism was the only trait exerting a meaningful impact on the capability of both people with dementia and their caregivers to ‘live well’ [30, 31]. However, to our knowledge no research has examined whether the Five-Factor personality traits have a meaningful impact on ‘living well’ in people with dementia and/or their caregivers longitudinally.

Longitudinal research is needed to further establish the role of personality in influencing the capability of people with dementia and their caregivers to ‘live well’. The present study utilizes longitudinal data from the IDEAL cohort. The aims of the study were to (a) describe the personality profiles of a large sample of people with dementia and their caregivers by classifying individuals as showing low, medium, or high levels of each of the five personality traits and (b) explore whether these personality trait groupings are related to meaningful variability in capability to ‘live well’ with the condition over two years.

## Methods

### Design

The present study used data from the first three timepoints of the IDEAL programme longitudinal cohort study [14], designed to investigate influences on ‘living well’ with dementia. The baseline assessment (timepoint 1; T1) was conducted between July 2014 and August 2016 with follow-up assessments conducted approximately 12 (timepoint 2; T2) and 24 (timepoint 3; T3) months later. At T1 1537 people with dementia together with 1277 caregivers were recruited through 29 National Health Service (NHS) sites across England, Scotland, and Wales. Inclusion criteria for people with dementia were, on entry to the study, a clinical diagnosis of dementia, living in the community, and a Mini-Mental State Examination [32] score of 15 or above indicating mild-to-moderate dementia. People with dementia were ineligible if they were unable to provide informed consent at T1, had co-morbid terminal illness, or there was known potential for home visits to pose a significant risk to researchers. People with dementia were invited to nominate a caregiver to take part in the study to act as an informant and to provide information on their own caregiving experiences. There were no specific inclusion or exclusion criteria for caregivers other than being willing and available to take part in the study. For the purposes of the study ‘caregiver’ was defined as the main family member or friend providing practical or emotional unpaid support to the person with dementia [31].

The IDEAL study received approval from the Wales Research Ethics Committee 5 (reference 13/WA/0405) and the Ethics Committee of the School of Psychology, Bangor University (reference 11684). The IDEAL study was registered with the United Kingdom Clinical Research Network (UKCRN), registration number 16593.

### Procedure

At all three timepoints participants were visited at home by a researcher from one of the 29 recruiting NHS sites. Informed consent was obtained prior to any study procedures. People with dementia were administered the assessment whilst participating caregivers self-completed their assessments. Selected measures from the IDEAL assessments were included in this analysis.

### Measures

#### Personality traits

At T1 only, people with dementia and caregivers completed the Mini-International Personality Item Pool (Mini-IPIP) [33], which contains 20 items from the International Personality Item Pool – Five Factor Model [34]. Four items assessed each of the five traits: neuroticism, extraversion, conscientiousness, agreeableness, and intellect and imagination - subsequently referred to as openness. Positively keyed items were rated on a scale from 1 to 5 and negatively keyed items were reverse coded providing a range of scores for each trait of 4–20; the higher the score the more pronounced the trait. These total scores were then used to categorize each participant as showing low, medium, or high levels of each trait based on a recent method [35]. Briefly, total scores for each trait were converted into z scores which were then converted into stanine scores. Stanine scores for each trait were then converted into the three groups so that stanine scores 1 to 3 formed the low group, stanine scores 4 to 6 formed the medium group, and stanine scores 7 to 9 formed the high group. This grouping method makes it possible to explore whether people with high, medium, or low levels of each trait displayed meaningful variability in capability to ‘live well’ over the two-year duration of the study. The Mini-IPIP was selected primarily due to its brevity as part of an extensive survey battery. While the validity of the Mini-IPIP for people with dementia has not been established, it has been used in studies of older people without dementia [36, 37]. These studies reported satisfactory internal consistency for the Mini-IPIP subscales (ranging between 0.60 and 0.82), albeit lower than studies using other Five-Factor personality measures [12, 38], likely due to fewer number of items per trait.

#### Living well

Subjective appraisals of ‘living well’ in people with dementia and their caregivers were assessed using individual measures of QoL, satisfaction with life, and well-being at all three timepoints. For the present study a single latent factor representing ‘living well’ was estimated from these measures.

For people with dementia QoL was assessed with the QoL in Alzheimer’s Disease scale (QoL-AD) [39]; the 13 items are each rated on a 1 to 4 scale, yielding a total score of 13–52. Caregiver QoL was assessed with the World Health Organization QoL-BREF(WHOQoL-BREF) [40] which contains 24 items assessing four QoL domains (physical, psychological, social, and environmental) and two single items assessing overall QoL and general health. As WHOQoL-BREF yields no overall total score, a QoL factor score was calculated as previously described [31]. People with dementia and caregivers completed the same measures of satisfaction with life and well-being. The 5-item Satisfaction with Life Scale (SwLS) [41] was used to assess satisfaction with life; each item is rated on a 1 to 7 scale providing a score of 5–35. Well-being was assessed using the 5-item World Health Organization-Five Well-being Index (WHO-5) [42]; each item is rated on a 0 to 5 scale providing a raw score from 0–25, which is converted to a percentage score (0-100). For all measures that comprise the ‘living well’ composite, higher scores indicate greater capability to ‘live well’.

#### Covariates

Scores on the following variables at T1 were used as covariates in the analysis.

##### **People with dementia**

Details of age, sex, socio-economic status (National Statistics Socio-economic classification-3; NS-SEC-3) [43] and diagnosis (Alzheimer’s disease, vascular dementia, mixed Alzheimer’s and vascular dementia, frontotemporal dementia, Parkinson’s disease dementia, dementia with Lewy bodies, or unspecified/other) were collected by the researchers. Dementia diagnostic information was collected from medical records. The Addenbrooke’s Cognitive Examination-III (ACE-III) [44] was administered to assess cognitive functioning and is scored out of 100, with higher scores indicating better cognitive function.

##### **Caregivers**

Details of age, sex, socio-economic status (NS-SEC-3), and caregiver status (spouse/partner vs. family/friend) were collected in the survey. Caregiver stress was assessed using the 15-item Relative Stress Scale [45]; this yields a total score out of 60, with higher scores indicating greater perceived stress.

### Statistical analysis

All analyses were based on version 6 of the IDEAL dataset and conducted using Mplus Version 8.2. A latent factor representing ‘living well’ was estimated from QoL-AD, SwLS, and WHO-5 scores for people with dementia. For caregivers, ‘living well’ was estimated from WHOQoL-BREF, SwLS, and WHO-5. SwLS was used as the marker, with the ‘living well’ factor taking on the same scale as SwLS (i.e., 5–35). Mean change over the three timepoints was examined using a latent growth curve model, comprising a mean intercept and slope, with random effects accounting for variation across individuals. Further details are provided in the Supplementary Appendix, Supplementary Table 1, and Supplementary Fig. 1. For the personality traits, the medium group was used as the reference. Associations of personality traits measured at T1 with the intercept (baseline) and slope (change over time) of ‘living well’ were investigated after adjusting for covariates.

Differences in ‘living well’ scores at baseline between the low and high groups for each personality trait were considered meaningful if the estimated effect sizes exceeded 1.5 [30]. In addition, a Reliable Change Index [46] was calculated, as previously described [47], to determine the reliability of any change over time in ‘living well’ and to account for measurement error.

#### Missing data

Where there were missing values on outcome measures, Mplus used the full information maximum likelihood estimator [48]. Multiple imputation of missing data on covariates was generated from Markov Chain Monte Carlo simulations [49]. Estimates from 25 imputed datasets were combined using Rubin’s rules [50].

## Results

### Cohort characteristics

Of the 1537 people with dementia and 1277 caregivers recruited to IDEAL at T1, only those that completed the mini-IPIP at T1 were included in the present study. In addition, where the caregiver recruited at baseline was replaced by another caregiver at T2 or T3, the replacement caregiver was excluded from analysis. Therefore, 1487 people with dementia and 1234 caregivers were included at T1, 1160 people with dementia and 947 caregivers at T2, and 836 people with dementia and 722 caregivers at T3.

For people with dementia just over half were male and just over half were diagnosed with Alzheimer’s disease. Over two-thirds of caregivers were female and were the co-resident spouse or partner of the person with dementia. The majority of people with dementia (93.9%) and caregivers (95.9%) classified themselves as white British. Characteristics of the sample and the reasons for withdrawal at each timepoint are summarized in Table 1.

**Table 1:** Characteristics of the sample and descriptive statistics for study variables

People with dementia	T1 (n = 1487)	T2 (n = 1160)	T3 (n = 836)	Caregiver	T1 (n = 1234)	T2 (n = 947)	T3 (n = 722)
	(N)	(N)	(N)		(N)	(N)	(N)
Withdrew/lost to follow-up	-	270	263	Withdrew/lost to follow-up	-	234	179
Did not take part at this timepoint	-	11	-	Did not take part at this timepoint	-	15	-
Died	-	46	72	Person with dementia died	-	36	61
				Caregiver died	-	2	0
	N (%)	N (%)	N (%)		N (%)	N (%)	N (%)
**Sex**				**Sex**			
Male	834 (56.1)	654 (56.4)	467 (55.9)	Male	377 (30.6)	291 (30.7)	227 (31.4)
Female	653 (43.9)	506 (43.6)	369 (44.1)	Female	857 (69.4)	656 (69.3)	495 (68.6)
Socio-economic status*				Socio-economic status*			
Class 1	613 (41.2)			Class 1	505 (40.9)		
Class 2	430 (28.9)			Class 2	431 (34.9)		
Class 3	415 (27.9)			Class 3	256 (20.7)		
Never worked/missing	29 (2.0)			Never worked/missing	42 (3.4)		
**Dementia diagnosis**				**Care recipient diagnosis**			
Alzheimer’s disease	829 (55.7)	647 (55.8)	480 (57.4)	Alzheimer’s disease	692 (56.1)	529 (55.9)	414 (57.3)
Vascular dementia	160 (10.8)	114 (9.8)	81 (9.7)	Vascular dementia	134 (10.9)	90 (9.5)	70 (9.7)
Mixed Alzheimer’s and vascular	315 (21.2)	260 (22.4)	182 (21.8)	Mixed Alzheimer’s and vascular	253 (20.5)	208 (22.0)	152 (21.1)
Frontotemporal dementia	53 (3.6)	39 (3.4)	31 (3.7)	Frontotemporal dementia	44 (3.6)	36 (3.8)	29 (4.0)
Parkinson’s disease dementia	41 (2.8)	33 (2.8)	16 (1.9)	Parkinson’s disease dementia	40 (3.2)	31 (3.3)	19 (2.6)
Dementia with Lewy bodies	49 (3.3)	39 (3.4)	27 (3.2)	Dementia with Lewy bodies	41 (3.3)	32 (3.4)	22 (3.0)
Unspecified dementia/other	40 (2.7)	28 (2.4)	19 (2.3)	Unspecified dementia/other	30 (2.4)	21 (2.2)	16 (2.2)
				**Caregiver status**			
				Spouse/partner	1013 (82.1)	798 (84.3)	613 (84.9)
				Family/friend	221 (17.9)	149 (15.7)	109 (15.1)
	M (SD), missing	M (SD), missing	M (SD), missing		M (SD), missing	M (SD), missing	M (SD), missing
**Mean age**	76.4 (8.48), 0	77.1 (8.38), 0	77.5 (8.46), 0	**Mean age**	69.1 (10.94), 0	70.3 (10.47), 0	71.0 (10.36), 0
**ACE-III**	69.4 (13.08), 96	66.5 (15.83), 102	64.8 (17.81), 108	**Stress**	19.15 (9.82), 54	21.8 (10.07), 63	23.1 (10.15), 46
Personality*				Personality*			
Neuroticism	10.2 (3.46), 24			Neuroticism	10.9 (3.15), 6		
Extraversion	11.7 (3.73), 17			Extraversion	12.1 (3.34), 10		
Conscientiousness	13.5 (3.04), 26			Conscientiousness	15.5 (2.66), 6		
Openness	12.8 (3.18), 47			Openness	13.2 (2.95), 22		
Agreeableness	15.8 (2.81), 19			Agreeableness	16.3 (2.61), 5		
**‘Living well’**				**‘Living well’**			
Quality of life	36.9 (5.89), 135	37.0 (5.90), 133	37.0 (5.60), 134	Quality of life (factor score)	0.2 (2.06), 23	0.1 (2.06), 46	-0.17 (2.07), 31
Satisfaction with life	26.2 (6.05), 37	26.3 (6.12), 68	26.3 (6.31), 87	Satisfaction with life	23.8 (6.48), 14	22.5 (6.80), 46	21.8 (6.58), 31
Well-being	61.2 (20.40), 21	60.8 (20.75), 49	61.4 (20.92), 68	Well-being	55.4 (19.70), 14	54.0 (20.54), 42	52.2 (20.30), 28

Note: ACE-III = Addenbrooke’s Cognitive Examination-III. For socio-economic status, class 1 = higher managerial, administrative, and professional occupations; class 2 = intermediate occupations; class 3 = routine and manual occupations. * Socio-economic status and personality were measured at T1 only

### Personality profiles and trait groupings

People with dementia had lower overall mean scores for each personality trait than caregivers. However, as standard deviations for people with dementia and caregivers intercept, this suggests that mean scores were generally equivalent; see Table 1. The score boundaries and descriptive statistics for the personality trait groupings are reported in Table 2. Responses indicate that, compared to the other traits, more people endorsed very high levels of the agreeableness trait, with 30% of people with dementia scoring 18–20 and 24.7% of caregivers scoring 19–20.

**Table 2:** Personality trait groupings and descriptive statistics

Trait	Group	People with dementia			Caregivers		
		Range	*n* (%)	Mean (SD)	Range	*n* (%)	Mean (SD)
Neuroticism	Low	4–7	321 (21.9)	5.7 (1.18)	4–8	280 (22.8)	6.6 (1.30)
	Medium	8–12	806 (55.1)	9.9 (1.42)	9–13	704 (57.3)	11.0 (1.37)
	High	13–20	336 (23.0)	15.0 (1.86)	14–20	244 (19.9)	15.3 (1.52)
Extraversion	Low	4–8	286 (19.5)	6.5 (1.39)	4–9	275 (22.5)	7.5 (1.41)
	Medium	9–14	833 (56.7)	11.4 (1.71)	10–14	653 (53.3)	12.1 (1.40)
	High	15–20	351 (23.9)	16.7 (1.55)	15–20	296 (24.2)	16.4 (1.32)
Conscientiousness	Low	4–11	373 (25.5)	9.7 (1.48)	6–13	273 (22.2)	11.8 (1.38)
	Medium	12–15	691 (47.3)	13.5 (1.10)	14–17	641 (52.2)	15.5 (1.10)
	High	16–20	397 (27.2)	17.3 (1.28)	18–20	314 (25.6)	18.8 (0.78)
Openness	Low	4–10	329 (22.8)	8.7 (1.52)	5–11	339 (28.0)	9.8 (1.38)
	Medium	11–15	824 (57.2)	12.9 (1.30)	12–15	610 (50.3)	13.3 (1.09)
	High	16–20	287 (19.9)	17.5 (1.42)	16–20	263 (21.7)	17.4 (1.35)
Agreeableness	Low	6–13	322 (21.9)	11.6 (1.40)	6–14	299 (24.3)	12.7 (1.49)
	Medium	14–17	706 (48.1)	15.7 (1.05)	15–18	627 (51.0)	16.5 (1.06)
	High	18–20	440 (30.0)	18.9 (0.81)	19–20	303 (24.7)	19.5 (0.50)

Note: SD = Standard Deviation

### Findings for people with dementia

#### Baseline

Adjusted model 1 shows that each of the five traits had a meaningful impact on ‘living well’ at baseline, with the difference in mean ‘living well’ scores between the low and high groups exceeding 1.5; see Table 3. For neuroticism, where lower scores are better, the high group had poorer ‘living well’ scores and the low group had better ‘living well’ scores. For the other four traits, where higher scores are better, the low group had poorer ‘living well’ scores and the high group had better ‘living well’ scores. The largest difference in mean ‘living well’ scores between the low and high groups was for neuroticism (5.6 points); see Supplementary Fig. 2.

**Table 3:** The relationship between personality trait groupings and ‘living well’ in people with dementia

Trait groupings	Unadjusted Model		Adjusted Model 1		Adjusted Model 2	
	Intercept	Slope	Intercept	Slope	Intercept	Slope
**Neuroticism**						
Low	2.02 (1.46–2.59)	0.03 (-0.28–0.34)	1.83 (1.28–2.38)	0.05 (-0.26–0.35)	1.83 (1.29–2.38)	0.05 (-0.26–0.35)
Medium	Ref	Ref	Ref	Ref	Ref	Ref
High	-3.99 (-4.57 – -3.42)	0.33 (0.02–0.64)	-3.76 (-4.33 – -3.19)	0.35 (0.04–0.67)	-3.76 (-4.33 – -3.19)	0.35 (0.03–0.67)
**Extraversion**						
Low	-2.19 (-2.83 – -1.56)	0.11 (-0.22–0.44)	-2.07 (-2.68 – -1.46)	0.14 (-0.19–0.47)	-2.07 (-2.68 – -1.46)	0.14 (-0.19–0.47)
Medium	Ref	Ref	Ref	Ref	Ref	Ref
High	1.52 (0.93–2.10)	0.11 (-0.19–0.41)	1.61 (1.05–2.18)	0.12 (-0.18–0.42)	1.62 (1.05–2.18)	0.12 (-0.18–0.42)
**Conscientiousness**						
Low	-2.00 (-2.58 – -1.41)	0.24 (-0.07–0.54)	-1.77 (-2.34 – -1.20)	0.22 (-0.08–0.53)	-1.77 (-2.33 – -1.20)	0.22 (-0.08–0.53)
Medium	Ref	Ref	Ref	Ref	Ref	Ref
High	1.75 (1.17–2.32)	-0.10 (-0.40–0.20)	1.63 (1.07–2.18)	-0.10 (-0.40–0.19)	1.64 (1.09–2.20)	-0.11 (-0.41–0.18)
**Openness**						
Low	-0.90 (-1.50 – -0.30)	0.18 (-0.13–0.48)	-0.90 (-1.49 – -0.32)	0.15 (-0.16–0.46)	-0.91 (-1.49 – -0.33)	0.15 (-0.16–0.46)
Medium	Ref	Ref	Ref	Ref	Ref	Ref
High	1.00 (0.36–1.64)	0.07 (-0.25–0.38)	0.90 (0.28–1.52)	0.05 (-0.27–0.37)	0.92 (0.30–1.54)	0.05 (-0.27–0.37)
**Agreeableness**						
Low	-0.84 (-1.47 – -0.21)	-0.28 (-0.60–0.05)	-0.75 (-1.36 – -0.14)	-0.26 (-0.59–0.07)	-0.77 (-1.38 – -0.16)	-0.26 (-0.58–0.07)
Medium	Ref	Ref	Ref	Ref	Ref	Ref
High	0.79 (0.22–1.36)	-0.01 (-0.30–0.27)	0.83 (0.27–1.38)	-0.01 (-0.30–0.28)	0.84 (0.29–1.40)	-0.01 (-0.30–0.27)

Note: 1442 people with dementia had outcome data for at least one timepoint and were included in the analysis. Adjusted Model 1 controls for age, sex, dementia diagnosis, and socio-economic status. Adjust Model 2 controls for Addenbrooke’s Cognitive Examination-III scores in addition to the covariates in Adjusted Model 1

### Longitudinal

‘Living well’ scores declined marginally by 0.16 points per year for people with dementia. A change of 7.1 points (‘living well’ takes on the scale of the SwLS score) was needed to signify reliable change. Therefore, there was no reliable association with ‘living well’ over time for any trait. While there was some evidence that ‘living well’ scores for those in the high neuroticism group declined to a lesser extent than those in the medium group, the numerical change was small, well below the Reliable Change Index, and was likely due to low ‘living well’ scores for this group at T1.

The unadjusted model and adjusted model 2 (further adjusted for ACE-III scores) provided similar estimates to adjusted model 1, indicating that for people with dementia neither background characteristics nor cognition influenced the relationship between personality and ‘living well’ at baseline or over time.

### Findings for caregivers

#### Baseline

Adjusted model 1 shows that neuroticism, conscientiousness, and extraversion had a meaningful impact on ‘living well’ scores at baseline, with the difference in mean ‘living well’ scores between the low and high groups exceeding 1.5. For extraversion and conscientiousness, the low group had poorer ‘living well’ scores and the high group had better ‘living well’ scores. For neuroticism, those with lower neuroticism had better ‘living well’ scores and vice versa, see Table 4. The largest difference in mean ‘living well’ scores between the low and high groups was for neuroticism (8.23 points); see Supplementary Fig. 3. The unadjusted model provided similar estimates. Adjusted model 2 indicated that, while the impact of neuroticism on ‘living well’ remained meaningful, when compared to adjusted model 1 caregiver stress attenuated the difference in ‘living well’ scores between the low and high groups to 5.21 points.

**Table 4:** The relationship between personality trait groupings and ‘living well’ in caregivers of people with dementia

Trait groupings	Unadjusted		Adjusted 1		Adjusted 2	
	Intercept	Slope	Intercept	Slope	Intercept	Slope
**Neuroticism**						
Low	3.92 (3.29–4.54)	-0.12 (-0.42–0.17)	3.62 (3.00–4.24)	-0.13 (-0.43–0.17)	2.44 (1.92–2.97)	-0.13 (-0.44–0.18)
Medium	Ref	Ref	Ref	Ref	Ref	Ref
High	-4.68 (-5.34 – -4.01)	0.30 (-0.04–0.63)	-4.61 (-5.28 – -3.95)	0.28 (-0.06–0.62)	-2.77 (-3.34 – -2.20)	0.30 (-0.06–0.66)
**Extraversion**						
Low	-0.90 (-1.61 – -0.18)	0.43 (0.13–0.74)	-1.07 (-1.70 – -0.38)	0.42 (0.10–0.73)	-0.84 (-1.39 – -0.30)	0.41 (0.09–0.73)
Medium	Ref	Ref	Ref	Ref	Ref	Ref
High	1.71 (1.01–2.40)	-0.00 (-0.29–0.29)	1.64 (0.96–2.32)	-0.03 (-0.32–0.27)	1.50 (0.97–2.03)	-0.06 (-0.36–0.24)
**Conscientiousness**						
Low	-2.46 (-3.16 – -1.76)	0.59 (0.29–0.89)	-2.34 (-3.02 – -1.65)	0.61 (0.30–0.91)	-1.65 (-2.19 – -1.10)	0.62 (0.31–0.93)
Medium	Ref	Ref	Ref	Ref	Ref	Ref
High	1.31 (0.64–1.98)	0.25 (-0.03–0.54)	1.44 (0.79–2.09)	0.27 (-0.01–0.56)	1.31 (0.80–1.83)	0.28 (-0.02–0.57)
**Openness**						
Low	-0.05 (-0.75–0.66)	0.35 (0.03–0.66)	-0.07 (-0.76–0.63)	0.34 (0.02–0.65)	-0.42 (-0.97–0.13)	0.33 (0.01–0.66)
Medium	Ref	Ref	Ref	Ref	Ref	Ref
High	0.95 (0.22–1.68)	0.04 (-0.27–0.35)	0.71 (-0.01–1.43)	0.05 (-0.27–0.36)	0.89 (0.33–1.46)	0.06 (-0.27–0.38)
**Agreeableness**						
Low	-0.53 (-1.24–0.18)	0.09 (-0.21–0.39)	-0.97 (-1.69 – -0.25)	0.05 (-0.26–0.37)	-0.66 (-1.23 – -0.10)	0.05 (-0.27–0.38)
Medium	Ref	Ref	Ref	Ref	Ref	Ref
High	-0.13 (-0.83–0.57)	-0.05 (-0.34–0.24)	0.17 (-0.52–0.85)	-0.06 (-0.36–0.23)	0.28 (-0.26–0.82)	-0.07 (-0.37–0.23)

Note: 1234 caregivers had outcome data for at least one timepoint and were included in the analysis. Adjusted Model 1 controls for caregiver status, age, sex, care recipient diagnosis, and socio-economic status. Adjusted model 2 controls for Relative Stress Scale scores in addition to the covariates in Adjusted Model 1

### Longitudinal

The decline in ‘living well’ score for caregivers was 0.73 points per year; this was greater than the decline for people with dementia over the same time period, but still represents minimal change. A change of 6.2 points was needed to signify reliable change; therefore, there was no reliable association between any of the traits and ‘living well’ scores over time. While there was some evidence that the ‘living well’ scores of those in the low groups for extraversion, conscientiousness, and openness declined to a lesser extent than those in the medium groups, the numerical differences were small, well below the Reliable Change Index, and for extraversion and conscientiousness this was likely due to ‘living well’ scores being low at T1. The unadjusted and adjusted 2 models provided similar estimates indicating that for caregivers, neither background characteristics nor stress affected the relationship between each personality trait and ‘living well’ over time.

## Discussion

The present study was the first to investigate whether personality traits influence capability to ‘live well’ over time, drawing on data from a large cohort of people with mild-to-moderate dementia and their caregivers living in Britain. The study had two major aims; the first was to describe the personality profiles of a large cohort of people with dementia and their caregivers, by classifying individuals into low, medium, or high groups for each of the Five-Factor traits [35], and the second was to investigate whether personality traits affect the capability of people with dementia and caregivers to ‘live well’ over two years. Proportions of people categorized into the low, medium, and high groups for each trait were largely similar for people with dementia and caregivers. The largest difference was for openness where there were fewer people with dementia in the low group compared to caregivers whereas there were more people with dementia in the middle group than caregivers; proportions in the high group were broadly similar. For ‘living well,’ findings suggest that each personality trait had a meaningful impact on how people with dementia rated their ability to ‘live well’ at each timepoint; people higher in neuroticism reported poorer capability to ‘live well’ than those lower in neuroticism while those with higher levels of the remaining four traits reported greater capability to ‘live well’ than those with lower levels of these traits. For caregivers, neuroticism, conscientiousness, and extraversion had a meaningful impact on how they rated their capability to ‘live well’, although these associations were attenuated by caregiver stress. Personality traits had no reliable effect on how ‘living well’ scores changed over time for either people with dementia or caregivers, in either the unadjusted models or the models adjusted for background characteristics and cognition for people with dementia or stress for caregivers. Indeed, cognition had no effect on either ‘living well’ or personality ratings, suggesting that personality ratings made by people with mild-to-moderate dementia are not affected by cognitive decline and may be considered reliable.

The present study builds on the limited evidence concerning the relationship between personality traits and ‘living well’ in people with dementia and their caregivers. Consistent with our earlier studies [30, 31] neuroticism remained the most robust trait associated with self-reported ‘living well’ for both people with dementia and caregivers. Findings therefore suggest that of the five personality traits neuroticism may be most likely to influence how people with dementia and caregivers rate their capability to ‘live well’. However, as the influence of neuroticism did not change over time and as ‘living well’ was largely stable for both groups [47, 51] the impact of neuroticism on ‘living well’ is unlikely to affect how people with dementia or caregivers rate their capability to ‘live well’ over time.

The finding that caregiver stress attenuated the relationship between neuroticism and ‘living well’ at baseline, and that this impact remained stable over time, is in line with prior reports of an association between neuroticism and caregiver stress [22, 23]. It is possible that caregivers in the present study who were higher in neuroticism were at greater risk of feeling overwhelmed by stressful caregiving experiences which in turn negatively impacted their ‘living well’ scores. This suggests that future studies investigating personality traits in caregivers of people with dementia should control for stress. It also indicates that interventions targeting maladaptive coping responses to stress could be one way to support caregivers who are high in neuroticism.

The mean personality trait scores for people with dementia and their caregivers were generally similar. In addition, standard deviations intersected with scores in a similarly aged sample of older people without dementia in Britain that also used the Mini-IPIP [37]. This is interesting for two reasons. Firstly, high neuroticism and low conscientiousness scores have been suggested as risk factors for dementia [18, 52], suggesting that the people with dementia in the present study should have had noticeably higher levels of neuroticism and lower levels of conscientiousness than the other two groups. That said, the aetiology of dementia is complex and understanding dementia risk necessitates consideration of numerous factors, in addition to personality, and their interactions across the life course. Secondly, neuroticism is associated with increased health vigilance, symptom reporting, and help seeking behavior [53–55]. Therefore, a person with high neuroticism who experiences early signs of cognitive and/or functional difficulties may be more likely to seek medical help and obtain a diagnosis than a person with lower neuroticism. Similarly, a caregiver with high neuroticism may be more likely to seek medical help and subsequently facilitate a diagnosis of dementia for the care recipient. That said, any impact of neuroticism on obtaining a diagnosis is likely to be most prominent in the earliest stages of dementia. As symptoms develop or fail to resolve, as in those with mild-to-moderate dementia included in the present study, help seeking is likely to occur regardless of a person’s neuroticism levels. This may therefore explain why people with dementia and their caregivers did not have notably higher neuroticism scores than the sample of older people without dementia.

In addition, no study included in a recent meta-analysis [18] used the Mini-IPIP to measure personality; indeed, most used the NEO Five-Factor Inventory (NEO-FFI) [56]. The Mini-IPIP was selected for IDEAL due to its brevity as part of an extensive survey battery, thus maximizing cohort retention. However, the items used to assess each Five-Factor trait vary depending on the personality measure used, which may partly explain discrepancies between studies. Neuroticism is broadly defined as a tendency towards experiencing negative emotions such as anger, fear, sadness, irritability, and self-consciousness [19]. However, the items that assess neuroticism in the Mini-IPIP include ‘I am relaxed most of the time’ and ‘I seldom feel blue’. These items represent states that may be difficult for caregivers of people with dementia to achieve [31, 45] and it is possible that caregivers would respond to these items in relation to their caregiving role rather than more generally. The items that assess conscientiousness in the Mini-IPIP include questions such as ‘I get chores done right away’ and ‘I often forget to put things back in their proper place’. Responses to these items may be measuring difficulties that are common in dementia rather than conscientiousness. An extension of the present study using, for example, the more comprehensive NEO-FFI would enable a greater exploration of personality as a multi-componential construct as few items in the NEO-FFI concern aspects of dementia-related or caregiver-related states.

Despite the potential limitations of the Mini-IPIP, the use of self-rated personality data is a key strength of the present study. Several earlier seminal studies in this field relied on current and retrospective informant ratings [19, 20] which is a useful approach since informants usually have intimate knowledge of the history of the person with dementia, but possibly could be influenced by recall bias, the current behavioral and psychological symptoms of the person with dementia, or holding an idealized view of the person prior to dementia onset [57]. Although researchers have suggested that self-ratings of personality in people with dementia are inaccurate, owing to lack of self-awareness [58], this is based on the magnitude of the discrepancy between self-ratings made by people with dementia and ratings provided by their informants. The main limitation with this approach is that it assumes that informant ratings are accurate which is problematic for the several reasons outlined above. Prior studies have established the validity of self-ratings of other subjective constructs such as QoL in people with mild-to-moderate dementia [29]. The usefulness of self-ratings and incorporating the voice and subjective viewpoints of people with mild-to-moderate dementia is now widely recognized [30]. A limitation of the present study, and prior research, is that each of the Five-Factor personality traits are considered individually. However, personality is not necessarily seen as a separate set of characteristics but might include the interaction between characteristics to define personality types, something that could be studied with large enough samples for analysis. Another limitation was that personality was only assessed at baseline; this was to reduce participant burden and because there was less time for assessment at subsequent timepoints. Previous research in older people without dementia found that personality was largely stable over a three-year interval [12]. However, it is possible that as dementia severity increases personality may change [21]. Given the aforementioned issues with obtaining personality data from informants, more research is needed to understand the durability of personality traits in people with dementia. This could be achieved with large longitudinal, prospective studies that obtain self- and informant ratings before dementia develops and follow the sample as dementia progresses to investigate similarities and/or changes in traits. An inherent challenge is that self-ratings may be harder to obtain as dementia progresses into the advanced stages and will likely require the use of different scales and methodologies [59] making comparisons to any previously collected data difficult. It is also possible that personality may change as a function of disease type. Frontotemporal dementia, for example, is associated with more pronounced personality changes at earlier stages of the disease than other dementia subtypes [21]. Dementia diagnosis was included as a covariate and there was little effect of dementia diagnosis on the analysis. That said, the number of people with rarer dementia subtypes was small, thus reducing statistical power for these subtypes. Furthermore, while the proportion of people from minority ethnic groups was consistent with British population estimates [60], numbers were small which limits the generalizability of the findings to these groups.

Notwithstanding these limitations and the need for further research, the findings of the present study have some important clinical implications in highlighting that people with dementia and caregivers with high levels of neuroticism could benefit from greater support or targeted interventions. However, it may not be practical to assess personality traits in clinical settings; the present study suggests that the Mini-IPIP may not be optimal for people with dementia or their caregivers, and the 60-item NEO Five-Factor Inventory may be too long. Further work could establish the validity, reliability, and suitability of a brief measure to assess personality traits in people with dementia and their caregivers.

## Conclusions

This study extends prior research by indicating that personality does not appear to influence how capability to ‘live well’ changes over time in people with dementia or their caregivers. Indeed, ‘living well’ was largely stable over the two years and personality did not alter this trajectory. Findings suggest that neuroticism in particular may negatively affect how people with dementia and caregivers rate their capability to ‘live well’. Future research utilizing a more optimal measure of personality for people with dementia and their caregivers over a longer timeframe would be of particular interest in corroborating and extending the findings of the present study.

## Electronic supplementary material

Below is the link to the electronic supplementary material.

SupplementaryMaterial 1

## Data Availability

IDEAL data were deposited with the UK data archive in April 2020. Details of how to access the data can be found here: https://reshare.ukdataservice.ac.uk/854293/.

## List of abbreviations

QoL: Quality of life
IDEAL: Improving the experience of Dementia and Enhancing Active Life
T1: Timepoint 1
T2: Timepoint 2
T3: Timepoint 3
NHS: National Health Service
UKCRN: United Kingdom Clinical Research Network
Mini-IPIP: Mini-International Personality Item Pool
QoL-AD: Quality of Life in Alzheimer’s Disease scale
WHOQoL-BREF: World Health Organization Quality of Life Scale-BREF
SwLS: Satisfaction with Life Scale
WHO-5: World Health Organization-Five Well-being Index
NS-SEC-3: The National Statistics Socio-economic classification-3
ACE-III: Addenbrooke’s Cognitive Examination-III
NEO-FFI: NEO Five-Factor Inventory
SD: Standard Deviation
