# Supplementary material for: The associations between personality traits and quality of life, satisfaction with life, and well-being over time in people with dementia and their caregivers: findings from the IDEAL programme
Source: BMC Geriatr. 2023 Jun 6;23:354. doi: 10.1186/s12877-023-04075-x (PMC10242791; doi:10.1186/s12877-023-04075-x)
Supplement: Supplementary file 1 — SupplementaryMaterial 1 [file 12877_2023_4075_MOESM1_ESM.docx]

**The associations between personality traits and quality of life, satisfaction with life, and well-being over time in people with dementia and their caregivers: findings from the IDEAL programme.**

**Supplementary Material**

**Supplementary Appendix**

To determine changes over time in how people with dementia (PwD) and their caregivers perceive their capability to ‘live well’, latent growth curve growth modelling (LGCM) was conducted in Mplus Version 8.2 [1] using the first three waves of IDEAL data (T1-T3). A LGCM comprises a measurement model which is then extended to a second order growth model allowing estimation of the mean intercept (baseline) and the mean slope (change over time) of living well, with random effects to account for variation across individuals [2].

The ‘living well’ measurement model involves building the latent factor ‘living well’ from measures of QoL-AD, SwLS, and WHO-5 taken each year by longitudinal confirmatory factor analysis (LCFA) for PwD, and WHOQoL-BREF, SwLS, and WHO-5 for caregivers. SwLS was selected as the marker variable with loading fixed to 1 at each time point, and the intercept fixed to zero to allow for model identification. The scale of ‘living well’ takes on the same scale as SwLS and the variance of each latent factor and covariance among latent factors are defined by SwLS [3]. The associations among QoL and WHO-5 were estimated relative to their association with SwLS. Variances were estimated for each subdomain indicator and autocorrelated errors specified and retained in the model to avoid misspecification [4]. A good model will have a Comparative Fit index (CFI) and Tucker-Lewis index (TLI) greater than 0.90, and a root mean square error of approximation (RMSEA) less than 0.08 [5]. As shown in Supplementary Tables 1 and 2, the unconstrained measurement model (configural model) is a good fit to the data for both PwD and caregiver, indicating that each factor is defined by the same variables and that the same general pattern of factor loadings hold across time [6, 7]. In order for meaningful comparisons to be made in an LCFA, the assumption of longitudinal measurement variance should be met [8, 9]. Three levels of measurement invariance were tested imposing additional restrictions at each step: metric invariance (constrained factor loadings across measurement occasions), scalar invariance (constrained factor loadings across measurement occasions and intercepts across time to be equal), and strict invariance (constrained factor loadings across measurement occasions, and intercepts and residual variances across time to be equal). Each level of measurement invariance was applied and model fit indices examined to ensure that the model fit does not weaken when each level of constraint was applied. Studies have suggested that CFI, RMSEA and standardized root mean squared residual (SRMR) are the most important indicators when it comes to testing measurement invariance, with strict cut offs of <0.01 change in CFI, <0.015 change in RMSEA, and <0.030 change in SRMR [9, 10]. Δ*X*^2^ was also examined but is sensitive to sample size so was not relied upon [9, 11, 12]. As shown in Supplementary Table 1, each more constricted model had minimal impact on fit indices compared with the previous model supporting that metric, scalar, and strict measurement invariance held. Further analyses were conducted with the strict invariance model.

The ‘living well’ factors defined in the LCFA model were used as indicators of the second-order growth curve where the intercept and slope factors of ‘living well’ are estimated, each with their associated mean and variance. The model diagram is shown in Supplementary Figure 1A for PwD, and Supplementary Figure 1B for caregivers. The intercept loadings were fixed to 1 for each latent intercept, and 0, 1 and 2 for time based on the yearly measurement occasions. Due to only having three timepoints a linear trend was assumed.

**Supplementary Tables and Figures**

**Supplementary Table 1.**

Testing factorial measurement invariance for PwD models (A) and caregiver models (B). The configural model has no constraints and the metric model has constrained factor loadings across each measurement occasion. The scalar model has constrained factor loadings across each measurement occasion and intercepts across time are set to be equal. The strict model has constrained factor loadings across measurement occasions and intercepts and variances across time are set to be equal. ΔCFI, ΔRMSEA, and ΔSRMR were determined to assess model fit. Δ*X*^2^ was examined but not relied upon.

1. People with Dementia

| **Model** | **BIC** | ***X*^2^(df)** | **CFI** | **TLI** | **RMSEA (95% CI)** | **SRMR** | **Δ*X*^2^(Δdf)/p** | **ΔCFI** | **ΔTLI** | **ΔRMSEA** | **ΔSRMR** |
| --- | --- | --- | --- | --- | --- | --- | --- | --- | --- | --- | --- |
| Configural | 64778 | 23.92(16) | 0.999 | 0.997 | 0.018 (0.000 – 0.033) | 0.014 |  |  |  |  |  |
| Metric | 64761 | 35.86(20) | 0.997 | 0.995 | 0.023 (0.010 – 0.035) | 0.034 | 11.94(4)/0.018 | 0.002 | 0.002 | 0.005 | 0.020 |
| Scalar | 64734 | 39.06(24) | 0.998 | 0.996 | 0.021 (0.007 – 0.032) | 0.032 | 3.20(4)/0.525 | 0.001 | 0.001 | 0.002 | 0.002 |
| Strict | 64707 | 55.45(30) | 0.996 | 0.995 | 0.024 (0.014 – 0.034) | 0.044 | 16.39(6)/0.012 | 0.002 | 0.001 | 0.003 | 0.012 |

1. Caregivers

| **Model** | **BIC** | ***X*^2^(df)** | **CFI** | **TLI** | **RMSEA (95% CI)** | **SRMR** | **Δ*X*^2^(Δdf)/p** | **ΔCFI** | **ΔTLI** | **ΔRMSEA** | **ΔSRMR** |
| --- | --- | --- | --- | --- | --- | --- | --- | --- | --- | --- | --- |
| Configural | 48713 | 35.42(16) | 0.997 | 0.994 | 0.031 (0.017 – 0.045) | 0.016 |  |  |  |  |  |
| Metric | 48693 | 44.69(20) | 0.997 | 0.994 | 0.032 (0.019 – 0.044) | 0.024 | 9.29(4)/0.054 | 0.000 | 0.000 | 0.001 | 0.008 |
| Scalar | 48693 | 86.44(26) | 0.991 | 0.988 | 0.043 (0.033 – 0.054) | 0.026 | 41.75(4)/0.001 | 0.006 | 0.002 | 0.002 | 0.003 |
| Strict | 48669 | 90.98(30) | 0.991 | 0.990 | 0.041 (0.031 – 0.050) | 0.029 | 4.54(4)/0.338 | 0.000 | 0.002 | 0.002 | 0.003 |

1. People with Dementia


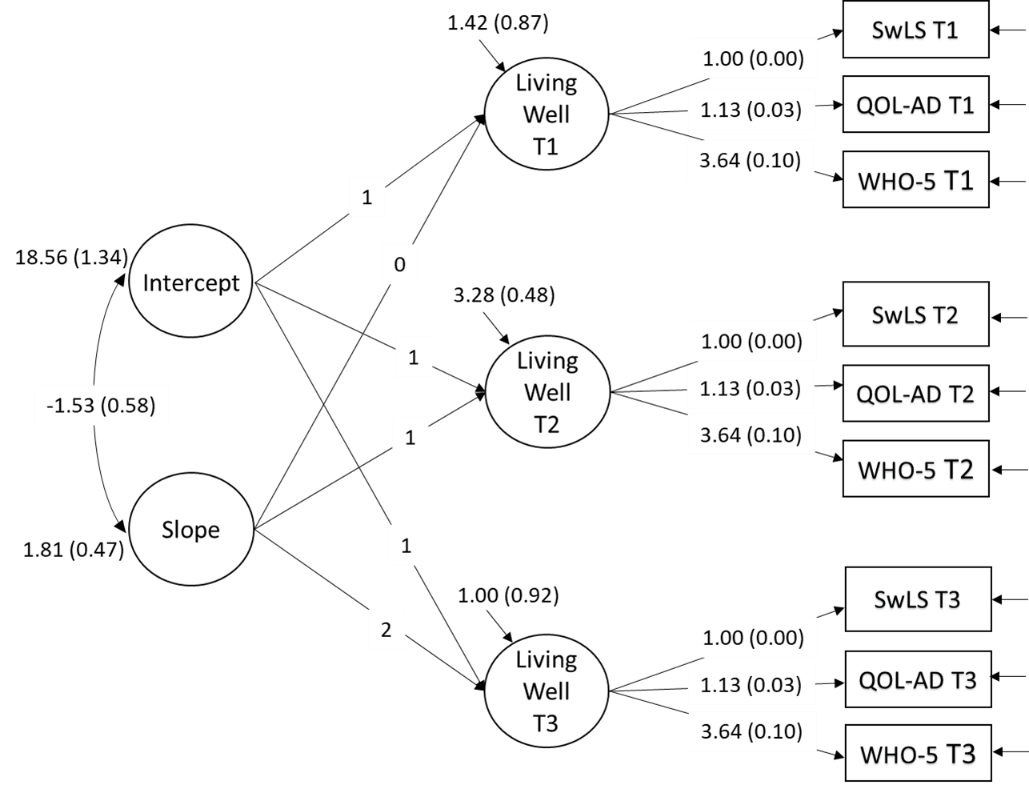


1. Caregiver


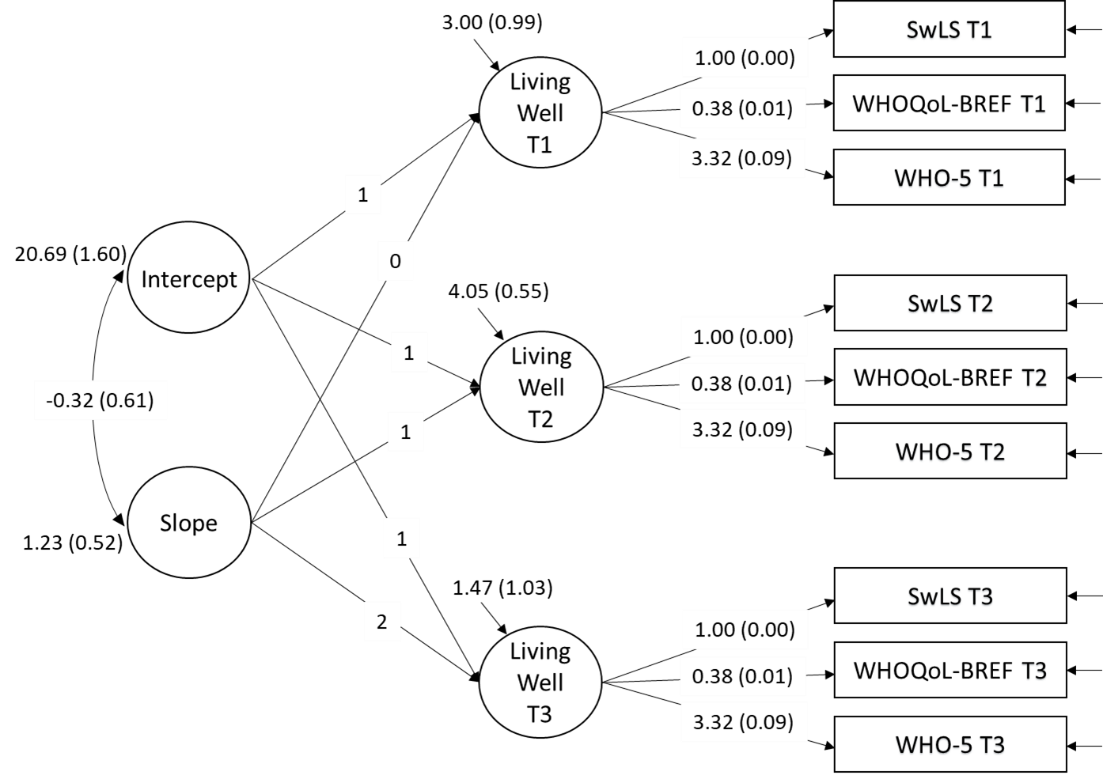


**Supplementary Figure 1.** The latent growth curve model involves the measurement model where the factors ‘living well’ T1 – ‘living well’ T3 are determined from measures of SwLS, QoL-AD, and WHO-5 at T1-T3 for PwD (A), and SwLS, WHOQOL-BREF, and WHO-5 at T1-T3 for caregivers (B). SwLS is used as the marker variable and loading fixed to 1. Intercept and slope latent factors are modelled from living well, with intercepts fixed to 1, and the occasions of the slopes fixed to 0, 1 and 2.

1. Neuroticism
2. Extraversion
3. Conscientiousness
4. Openness
5. Agreeableness

**Supplementary Figure S2.** Visualization of the mean intercepts and slopes for ‘living well’ by levels of (A) neuroticism, (B) extraversion, (C) conscientiousness, (D) openness, and (E) agreeableness in PwD. Graphs are representative of PwD that are female, 75 years old, have Alzheimer’s disease, and are in the middle SES category. *Note.* For neuroticism, low scores are better. For the other traits high scores are better.

1. Neuroticism
2. Extraversion
3. Conscientiousness
4. Openness
5. Agreeableness

**Supplementary Figure S3.** Visualization of the mean intercepts ad slopes for ‘living well’ by levels of (A) neuroticism, (B) extraversion, (C) conscientiousness, (D) openness, and (E) agreeableness in caregivers. Graphs are representative of caregivers that are female, 70 years old, caring for someone with Alzheimer’s disease, and are in the middle SES category. *Note.* For neuroticism, low scores are better. For the other traits high scores are better.

**References**

1. Muthén LK, Muthén BO. Mplus User's Guide. 8th ed: Muthén & Muthén; 1998-2017.

2. Wickrama KAS, Lee TK, O'Neal CW, Lorenz FO. Higher-order growth curves and mixture modeling with Mplus. A practical Guide: Routledge; 2016.

3. Brown TA. Confirmatory factor analysis for applied research. New York, NY: The Guilford Press; 2006.

4. Little TD. Longitudinal structural equation modelling. New York, NY: The Guilford Press; 2013.

5. Hu L, Bentler PM. Cutoff criteria for fit indexes in covariance structure analysis: Conventional criteria versus new alternatives. Structural Equation Modeling. 1999;6(1):1-55. <https://doi.org/10.1080/10705519909540118>.

6. Millsap RE, Cham H. Investigating factorial invariance in longitudinal data. In: Laursen B, Little TD, Card NA, editors. Handbook of developmental research methods: The Guilford Press; 2012. p. 109-26.

7. Millsap RE, Olivera-Aguilar M. Investigating measurement invariance using confirmatory factor analysis. In: Hoyle RH, editor. Handbook of structural equation modeling: The Guilford Press; 2012. p. 380-92.

8. Byrne BM, Watkins D. The issue of measurement invariance revisited. Journal of Cross-Cultural Psychology. 2003;34(2):155-75. <https://doi.org/10.1177/0022022102250225>.

9. Chen FF. Sensitivity of goodness of fit indexes to lack of measurement invariance. Structural Equation Modeling. 2007;14(3):464-504. <https://doi.org/10.1080/10705510701301834>.

10. Cheung GW, Rensvold RB. Evaluating goodness-of-fit indexes for testing measurement invariance. Structural Equation Modeling. 2002;9(2):233-55. <https://doi.org/10.1207/S15328007SEM0902_5>.

11. Schermelleh-Engel K, Moosbrugger H, Müller H. Evaluating the fit of structural equation models: tests of significance and descriptive goodness-of-fit measures. Meth Psychol Res. 2003;8(2):23-74.

12. Kline RB. Principles and practice of structural equation modeling. 3rd ed: The Guilford Press; 2011.
